# Construction of a prognostic risk assessment model for lung adenocarcinoma based on Integrin β family‐related genes

**DOI:** 10.1002/jcla.24419

**Published:** 2022-04-11

**Authors:** Yuanlin Wu, Linhai Fu, Bin Wang, Zhupeng Li, Desheng Wei, Haiyong Wang, Chu Zhang, Zhifeng Ma, Ting Zhu, Guangmao Yu

**Affiliations:** ^1^ 74682 Department of Thoracic Surgery Shaoxing People's Hospital Shaoxing China

**Keywords:** ITGB, lung adenocarcinoma, NMF, nomogram, prognostic model

## Abstract

**Background:**

Integrin β (ITGB) superfamily plays an essential role in the intercellular connection and signal transmission. It was exhibited that overexpressing of ITGB family members promotes the malignant progression of lung adenocarcinoma (LUAD), but the relationship between ITGB superfamily and the LUAD prognosis remains unclear.

**Methods:**

In this study, the samples were assigned to different subgroups utilizing non‐negative matrix factorization clustering according to the expression of ITGB family members in LUAD. Kaplan–Meier (K‐M) survival analysis revealed the significant differences in the prognosis between different ITGB subgroups. Subsequently, we screened differentially expressed genes among different subgroups and conducted univariate Cox analysis, random forest feature selection, and multivariate Cox analysis. 9‐feature genes (FAM83A, AKAP12, PKP2, CYP17A1, GJB3, TMPRSS11F, KRT81, MARCH4, and STC1) in the ITGB superfamily were selected to establish a prognostic assessment model for LAUD.

**Results:**

In accordance with the median risk score, LUAD samples were divided into high‐ and low‐risk groups. The receiver operating characteristic (ROC) curve of LUAD patients’ survival was predicted via K‐M survival curve and principal component analysis dimensionality reduction. This model was found to have a favorable performance in LUAD prognostic assessment. Gene Ontology (GO) and Kyoto Encyclopedia of Genes and Genomes (KEGG) analyses of differentially expressed genes between groups and Gene Set Enrichment Analysis (GSEA) of intergroup samples confirmed that the high‐ and low‐risk groups had evident differences mainly in the function of extracellular matrix (ECM) interaction. Risk score and univariate and multivariate Cox regression analyses of clinical factors showed that the prognostic model could be applied as an independent prognostic factor for LUAD. Then, we draw the nomogram of 1‐, 3‐, and 5‐year survival of LUAD patients predicted with the risk score and clinical factors. Calibration curve and clinical decision curve proved the favorable predictive ability of nomogram.

**Conclusion:**

We constructed a LUAD prognostic risk model based on the ITGB superfamily, which can provide guidance for clinicians on their prognostic judgment.

## Graphical abstract

Flowchart presenting the process of constructing prognostic model in this study.

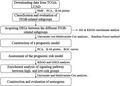

## 1 INTRODUCTION

The number of cancer patients is increasing year by year worldwide, and the patients tend to be younger. According to the latest data from the National Cancer Registry, lung cancer remains one of the cancers with the highest morbidity and mortality.^1^ Non‐small cell lung cancer (NSCLC) accounts for 80%‐85% of total lung cancer, while lung adenocarcinoma (LUAD) is the main histological subtype of NSCLC.^2^ The survival time of early LUAD patients can be prolonged by surgical treatment. However, due to the lack of specific clinical symptoms in the early stage, the opportunity for surgical treatment has been lost because of local infiltration or distant metastasis. The treatment technology for LUAD has currently been improved a lot. In addition to surgical resection, comprehensive treatments including radiotherapy and chemotherapy are also the main therapeutic methods for LUAD. However, the 5‐year survival rate of patients is still lower than that of most cancers (https://seer.cancer.gov/statfacts/). In recent years, despite the new hope for LUAD patients with target therapy, the prognosis is still not satisfactory.^3^, ^4^ Thus, biomarkers are utilized to identify the high‐risk patients with poor prognosis, which provides underlying therapeutic targets for LUAD treatment and improves the prognosis of LUAD patients, contributing to disease management and treatment.

Integrin β (ITGB) superfamily is a member of the integrin superfamily, which contains eight subtypes. Integrin is a kind of transmembrane heterodimer of somatic adhesion molecules that can provide connections and mediate interactions between cells and cells, cells, and extracellular matrixes (ECMs).^5^ ITGB superfamily also plays an initial role in the regulation of various cellular activities, including proliferation, carcinogenesis, and immune response.^6^ Puerkaiti et al.^7^ demonstrated that ITGB2 can enhance tumor progression and affect patients’ prognosis via inhibiting the identification and immune response of the immune system to tumor cells in triple‐negative breast cancer. Wu et al.^8^ revealed that inhibiting the expression of ITGB3 in gastric cancer can repress gastric cancer cells to migrate and invade. While ITGB1 was indicated by Li et al.,^9^ it induces radioresistance by affecting DNA repair and YAP1‐induced epithelial–mesenchymal transition in NSCLC. Zhu et al.^10^ proposed that the high expression of ITGB1 in NSCLC shortens the overall survival (OS) of patients. Wu et al.^11^ proposed that ITGB4 can be applied as the diagnostic biomarker for both LUAD and lung squamous cell carcinoma, ITGB8 can be used as the diagnostic biomarker for lung squamous cell carcinoma, and ITGB4 can also serve as an underlying prognostic biomarker for LUAD. However, the potential biological functions of ITGB5 and ITGB7 were scarcely understood. The above studies exhibited that the ITGB family is correlated with the malignant progression and prognosis of tumors. Therefore, the exploration of the influences of ITGB‐related genes on LUAD contributes to the prognostic assessment and the mining of potential biomarkers.

The prognostic effects of the ITGB superfamily on LUAD remain unsolved. We applied LUAD‐related mRNA expression data from the public databases to classify LUAD samples into subgroups according to the gene expression profiles of ITGB superfamily members. Then, based on the differentially expressed genes in varying subgroups, a prognostic risk assessment model related to ITGB superfamily for LUAD was established, in order to provide some references for screening potential biomarkers of LUAD patients and clinicians’ prognostic judgment.

## 2 MATERIALS AND METHODS

### 2.1 Data downloading

mRNA expression data and corresponding clinical information (age, survival, tumor staging, etc.) of LUAD patients were accessed from The Cancer Genome Atlas (TCGA) database (https://portal.gdc.cancer.gov/), including 535 LUAD samples and 59 normal samples.

### 2.2 Classification and evaluation of ITGB‐related subgroups

First, the samples with survival time of more than 30 days were screened from LUAD samples for subsequent analysis. Non‐negative matrix factorization (NMF) method was adopted for clustering analysis of samples based on the gene expression of ITGB8. The optimal cluster number was determined according to the area curve of NMF cophenetic, and the LUAD samples in the dataset were divided into subgroups.^12^ “factoextra” package (CRAN—Package factoextra (r‐project.org)) was utilized for principal components analysis (PCA) on ITGB‐related subtypes to verify the clustering. The survival curves of different subtypes were drawn applying the “survival” package.^13^

### 2.3 Screening of ITGB‐related prognostic genes and construction of a prognostic model

Differential analysis was performed on the genes in different ITGB‐related subgroups employing edgeR package^14^ with |logFC| > 1 and FDR < 0.05 as the standard to screen the differentially expressed genes in diverse subgroups. The clusterProfiler package^15^ was applied to conduct Gene Ontology (GO) enrichment and Kyoto Encyclopedia of Genes and Genomes (KEGG) signaling pathway analyses on all differentially expressed genes. The differentially expressed genes in different subgroups were analyzed by univariate Cox regression with the survival package, and the mRNA associated with prognosis was screened with *p *< 0.001 as the standard. The samples were randomized into training set and validation set at a ratio of 7:3 using the “caret” package. The “randomForestSRC” package was employed to conduct iterative elimination screening (ntree=1000, nrep=50) for prognostic mRNAs in the training set, and the optimal prognostic genes were obtained. The survival package was ultimately adopted to conduct multivariate Cox analysis on the mRNAs obtained in the previous step, followed by the obtaining of the ITGB family‐related prognostic genes and the construction of the risk assessment model.

### 2.4 Assessment of the prognostic risk model

In accordance with the expression level and risk coefficient of each feature in the samples, the risk score of each sample in the training set was calculated, and the patient samples were assigned to groups with the median risk score as the critical value. Then, PCA dimensionality reduction was conducted on different risk groups applying the factoextra package. The survival curves were drawn utilizing the survival package. In order to verify the effectiveness of the risk assessment model, the timeROC package was applied to draw receiver operating characteristic (ROC) curve, and the area under ROC curve (AUC) of 1‐, 3‐, and 5‐year OS of LUAD patients was calculated. Finally, the same verification was applied in the validation set.

### 2.5 Enrichment analysis of signaling pathway between high‐ and low‐risk groups

The limma package was employed to conduct the differential expression analysis on mRNAs in high‐ and low‐risk groups (|logFC| > 1, *p*_adj_ < 0.05). The enrichment of functions and pathways of the differentially expressed genes was analyzed on the online website Metascape (https://metascape.org/gp/index.html#/main/step1). Gene Set Enrichment Analysis (GSEA) was then adopted to perform KEGG pathway analysis on samples in two risk groups.

### 2.6 Construction and evaluation of nomogram

To investigate the independence of the ITGB superfamily‐related prognostic risk assessment model constructed in this study, univariate and multivariate Cox analyses were performed with risk score as a prognostic feature combined with other clinical factors (age, sex, TNM stage, and tumor stage). Combined with clinical factors and risk scores, the nomogram was generated to predict the 1‐, 3‐, and 5‐year OS of LUAD patients using the rms package.^16^ To evaluate the consistency between the actual survival and predicted survival of the nomogram, calibration curves were further drawn to measure the reliability of the model.

Subsequently, the method provided by Memorial Sloan‐Kettering Cancer Center (https://www.mskcc.org/departments/epidemiology‐biostatistics/biostatistics/decision‐curve‐analysis, MSKCC) was utilized to draw nomogram to predict the decision curves for 1‐, 3‐, and 5‐year OS of LUAD patients,^17^ thereby verifying the predictive performance of the nomogram.

## 3 RESULTS

### 3.1 Classification of ITGB gene‐related subgroup

**FIGURE 1:**
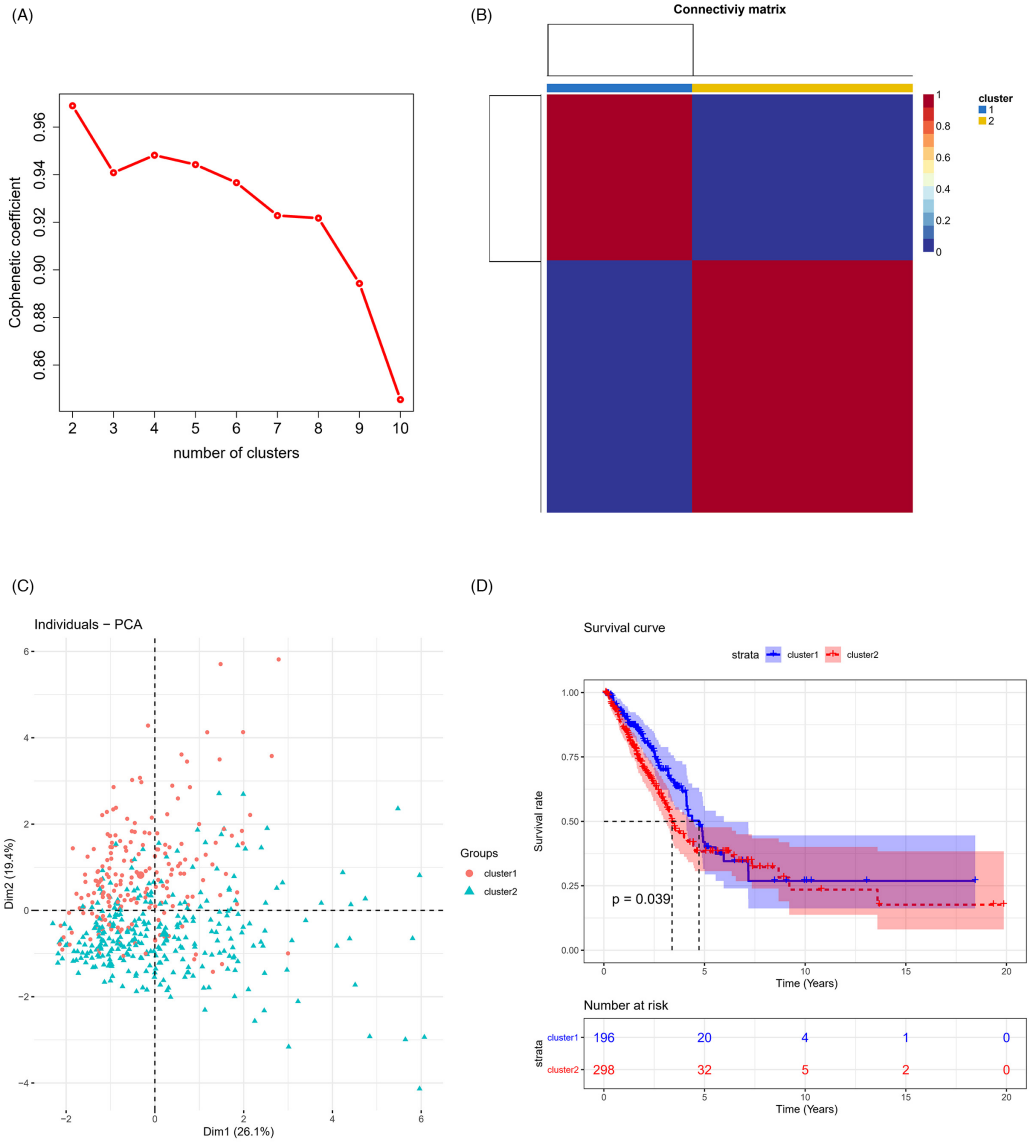
LUAD is divided into ITGB‐related subgroups based on the NMF model. (A) Area curve of NMF cophenetic at different *k* values; (B) 493 LUAD patients were divided into 2 ITGB‐related subgroups; (C) PCA dimensionality reduction analysis among ITGB‐related subgroups; (D) K‐M survival analysis among ITGB‐related subgroups (*p < 0.05)

First, the data in TCGA‐LUAD dataset were preprocessed, and 493 LUAD samples with survival time over 30 days and complete clinical information were screened. NMF method was applied to analyze the samples based on the ITGB expression profile. The area curve of NMF cophenetic was adopted to visualize the non‐negative matrix decomposition clustering analysis, with K representing the number of subgroups obtained by clustering. The results implicated that the model clustering was the most stable when *k* = 2, and LUAD samples were assigned to two subgroups cluster1 and cluster2 (Figure 1A,B). PCA dimensionality reduction in the two subgroups indicated that the two kinds of samples could be distinguished by the ITGB gene expression pattern (Figure 1C). The results of the survival analysis of two subgroups demonstrated the significant differences in the survival of samples between ITGB‐related subgroups (Figure 1D). Taken together, due to the notable correlation between the expression pattern of ITGB family members and LUAD prognosis, ITGB possessed a certain prognostic value.

**FIGURE 2:**
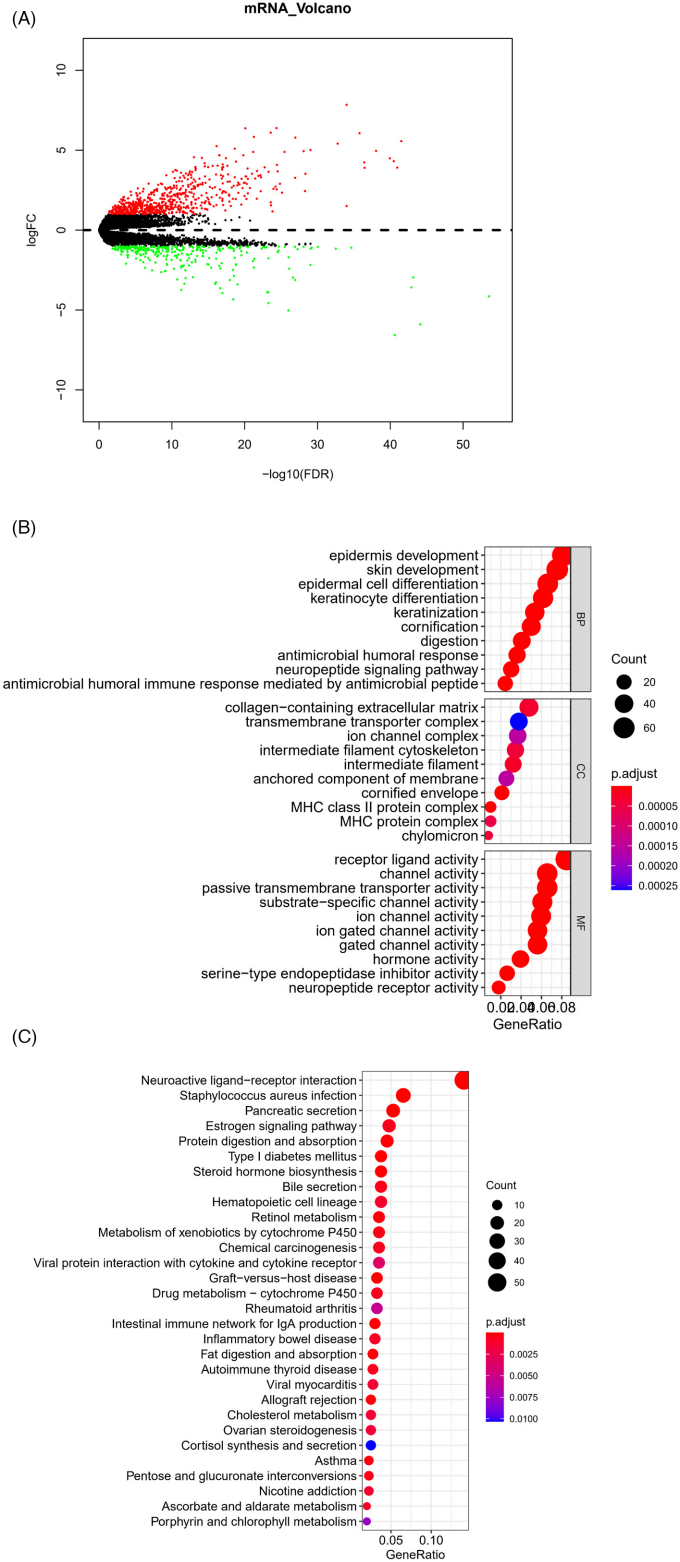
Differentially expressed genes and their involved functional pathways among ITGB‐related subgroups. (A) Volcano plot of differentially expressed genes in different ITGB subgroups (red: upregulated genes, green: downregulated genes); Bubble diagram of (B) GO enrichment analysis and (C) KEGG enrichment analysis of differentially expressed genes in ITGB subgroups

Due to the worse OS status of cluster2 than that of cluster1, we conducted differential expression analysis. Nine‐hundred and ninty‐six differentially expressed genes were totally found, with 707 upregulated genes and 289 downregulated genes (Figure 2A). Enrichment analyses were performed on these 996 genes. GO analysis confirmed that these differentially expressed genes were mainly enriched in the antimicrobial humoral response, neuropeptide signaling pathway, ion channel complex, MHC protein complex, receptor‐ligand activity, and other biological functions (Figure 2B). In addition, KEGG analysis demonstrated that these differentially expressed genes were mainly enriched in the biological pathways, such as estrogen signaling pathway and retinol metabolism (Figure 2C). Taken together, the functional differences between ITGB‐related subgroups were found to be enriched in immune signal regulation, tumor progression regulation, and other related pathways.

### 3.2 Construction of a prognostic model based on ITGB‐related 9‐feature genes

**FIGURE 3:**
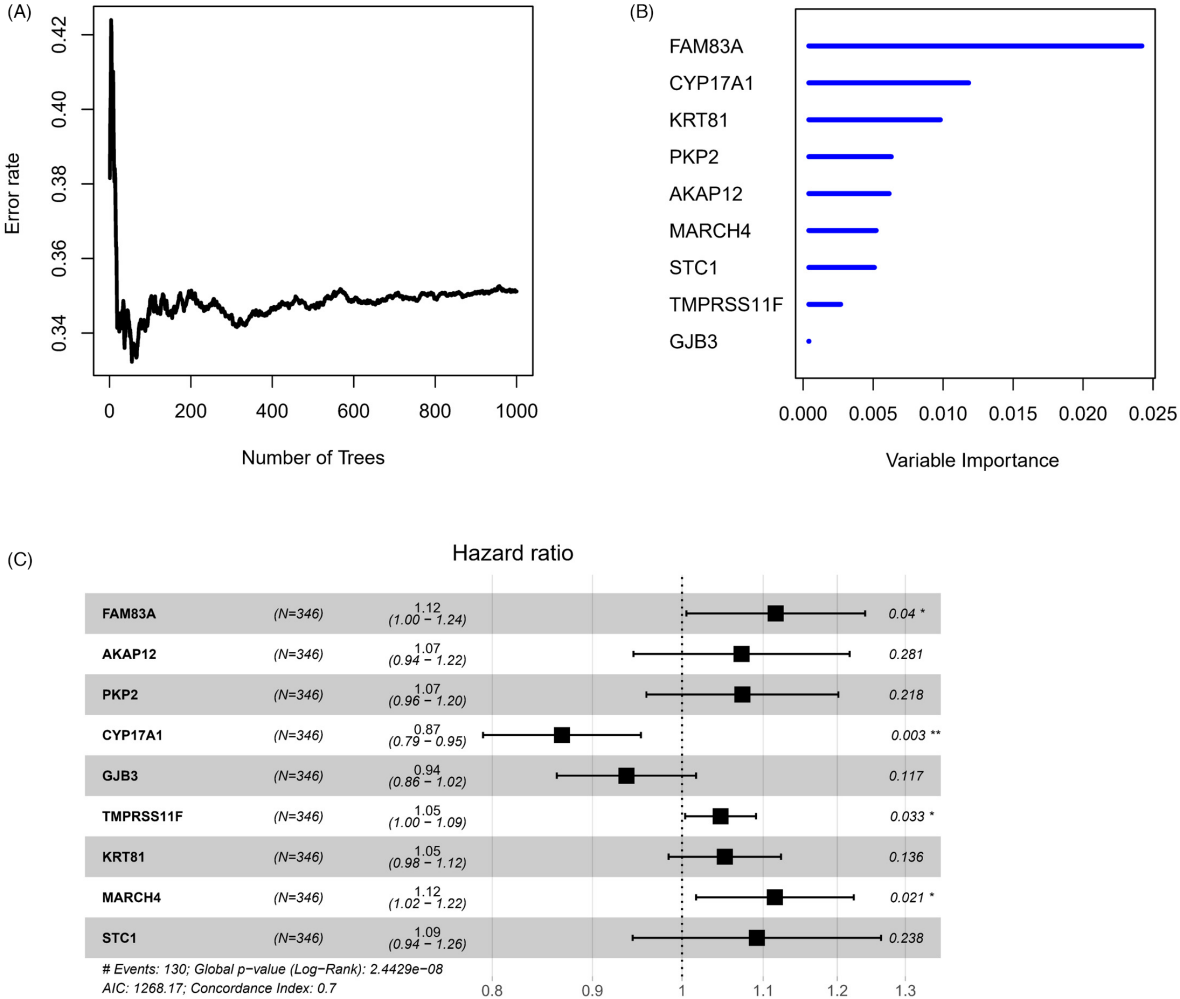
Construction of ITGB‐related prognostic model. (A) The relationship between error rate and the number of feature genes in random forest feature selection; (B) The importance sequencing of the screened 9 prognostic genes; (C) Forest map of multivariate Cox regression of 9‐feature genes (*p < 0.05, **p < 0.01)

Fifty LUAD prognosis‐related mRNAs were screened from differentially expressed genes in ITGB subgroups through univariate Cox regression analysis, with *p *< 0.001 as the screening condition (Table S1). Thereafter, the dataset was randomly divided into the training set and validation set at a ratio of 7:3. Random Forest method was used to carry out the feature selection on the training set based on the relationship between the error rate and the number of genes in the classification tree. The results exhibited that when the feature gene number was 9, error rate was 0.3512. Thereafter, with the increase in gene number, error rate did not decrease notably (Figure 3A,B). Hence, these 9 genes were selected for multivariate Cox analysis, thereby obtaining 9 optimal prognostic genes (FAM83A, AKAP12, PKP2, CYP17A1, GJB3, TMPRSS11F, KRT81, MARCH4, and STC1). The prognostic risk assessment model was risk score = 0.110* FAM83A + 0.070* AKAP12 + 0.071* PKP2 − 0.141* CYP17A1 − 0.065* GJB3 + 0.045* TMPRSS11F + 0.050* KRT81 + 0.109* MARCH4 + 0.088* STC1 (Figure 3C). The samples were assigned to low‐ and high‐risk groups by median risk score.

### 3.3 ITGB‐related prognostic model has a favorable predictive performance

**FIGURE 4:**
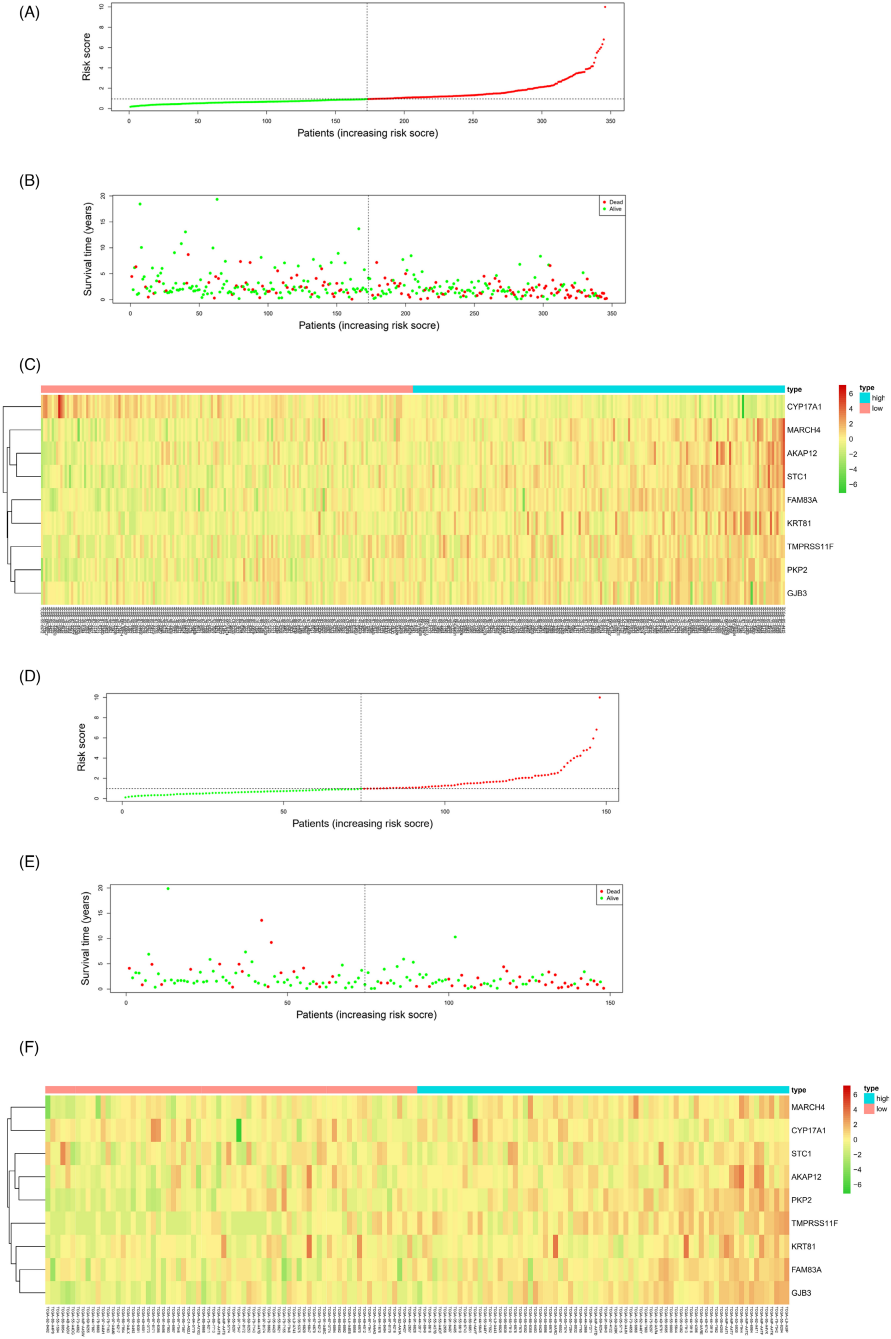
Survival of samples and expression of feature genes in high‐ and low‐risk groups. Risk score distribution chart of LUAD patients in the (A) training set and (D) validation set, with red displaying samples in the high‐risk group and green displaying samples in the low‐risk group; survival curves of LUAD patients in the (B) training set and (E) validation set obtained by risk score, with red representing the dead samples and green representing the living samples; Heatmap of 9‐feature gene expression in two risk groups in the (C) training set and (F) validation set

According to the clinical information of patients in different risk groups, the survival distribution map of LUAD patients was drawn. It could be observed that with the increase in the risk score of the training set samples, the number of LUAD deaths in the high‐risk group gradually increased and the survival time gradually shortened (Figure 4A,B). Combined with the heatmap of 9‐feature gene expression in two risk groups, it was indicated that in the training set, only CYP17A1 was evidently highly expressed in the high‐risk group (Figure 4C), and the results of the validation set were consistent with that of the training set (Figure 4D–F).

**FIGURE 5:**
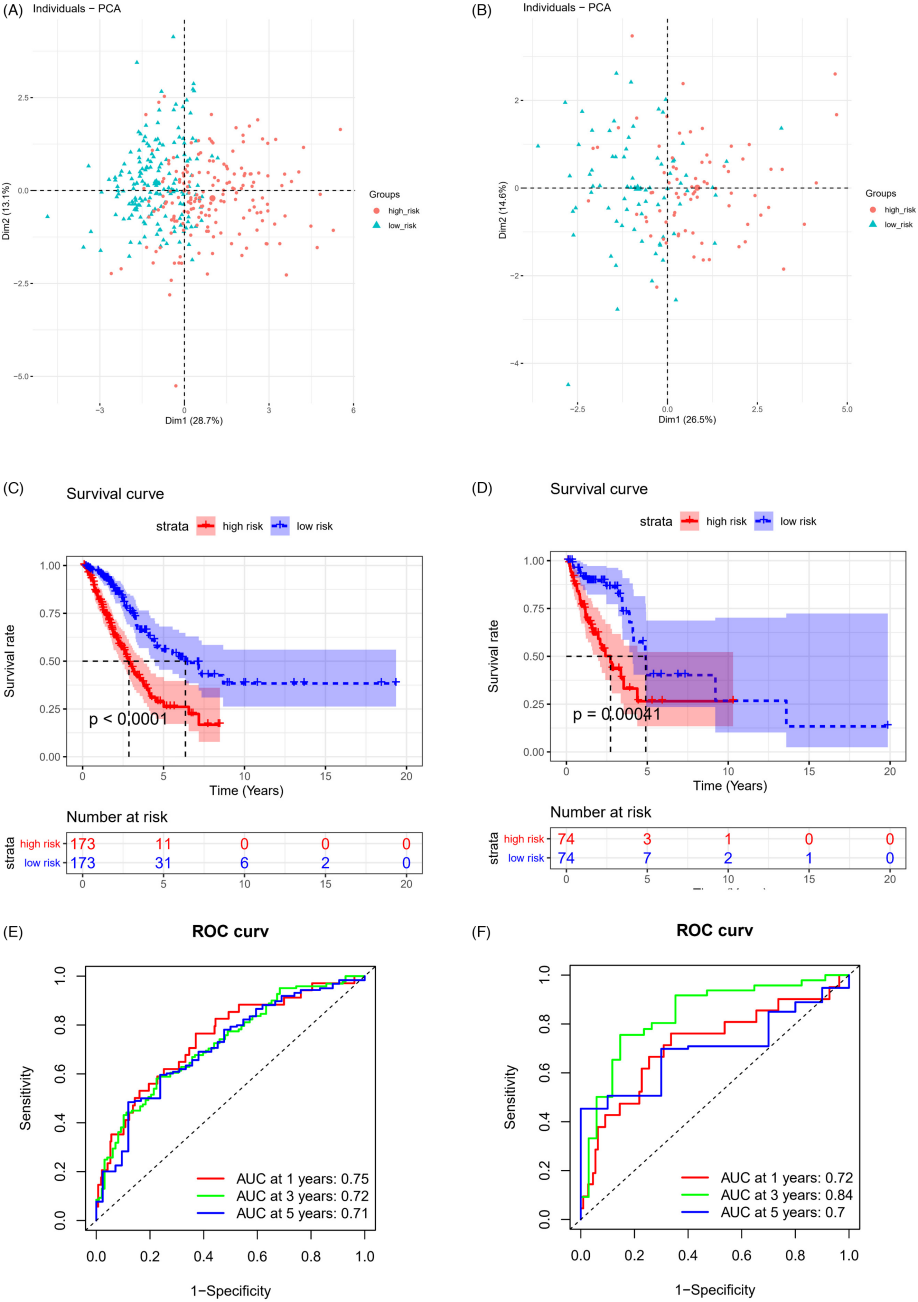
Assessment of predictive capacity of 9‐feature gene‐based prognostic model. PCA dimensionality reduction analysis of samples in the high‐ and low‐risk groups in the (A) training set and (B) validation set, with red indicating the high‐risk group and cyan indicating the low‐risk group; K‐M survival curves of patients in two risk groups in the (C) training set and (D) validation set, with red representing the high‐risk group and blue representing the low‐risk group; ROC curves of 9‐feature gene‐based prognostic model in the (E) training set and (F) validation set

PCA dimensionality reduction analysis was performed on the samples of the high‐risk group in the training set based on the 9‐feature genes, and the two risk groups of samples could be clearly distinguished (Figure 5A). Similar results were obtained in the validation set (Figure 5B). Subsequently, survival curves were drawn for the samples of two risk groups in the training set and validation set. It was observed that the survival rate of the high‐risk group was lower than that of the low‐risk group, indicating that the high‐risk group had a worse prognosis (Figure 5C,D). TimeROC curve hinted that 1‐, 3‐, and 5‐year AUC values predicted by the model in the training set were 0.75, 0.72, and 0.71, respectively (Figure 5E). While in the validation set, 1‐, 3‐, and 5‐year AUC values were 0.72, 0.84, and 0.7, respectively (Figure 5F). In summary, the 9‐feature gene prognostic risk assessment model could predict the survival of LUAD patients to a certain extent.

### 3.4 Different signaling pathways in the high‐ and low‐risk groups

**FIGURE 6:**
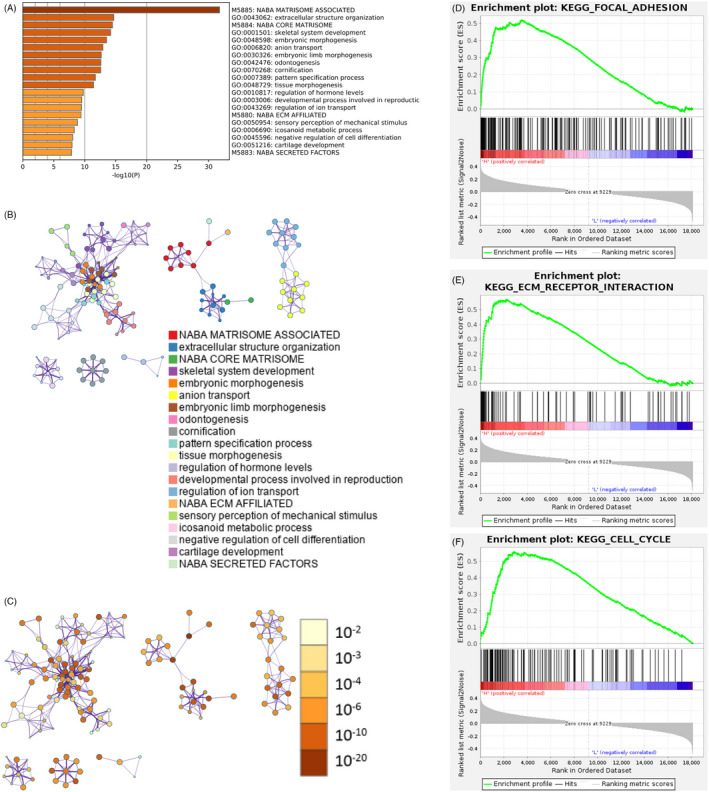
Differentially activated signaling pathways between high‐ and low‐risk groups. (A) Bar chart of *p*‐value distribution of biological processes and functional enrichment of differentially expressed genes in two risk groups in the training set. The horizontal axis represents the number of enriched genes; (B) ID cluster diagram and (C) *p*‐value cluster diagram of functional enrichment items of differentially expressed genes in the high‐ and low‐risk groups in the training set; Enrichment of two risk groups in (D) FOCAL_ADHESION gene set, (E) ECM_RECEPTOR_INTERACTION gene set and (F) CELL_CYCLE gene set

Differential expression analysis was performed on genes in two risk groups, and 936 differentially expressed genes were obtained. Enrichment analysis of differentially expressed genes in Metascape illustrated that these genes were mainly associated with NABA MATRISOME ASSOCIATED, anion transport, Processes regulation of hormone levels, and other biological functions (Figure 6A–C). GSEA software was then applied to conduct KEGG analysis on the differentially expressed genes in two risk groups. Evident differences were shown in the activation levels of signaling pathways, such as FOCAL_ADHESION, ECM_RECEPTOR_INTERACTION, and CELL_CYCLE between two groups (Figure 6D–F). Based on the results of the above functional analyses, differentially expressed genes were demonstrated to be enriched in ECM interaction and cell cycle regulation in two risk groups, suggesting that changes in these functions might be internal factors affecting the prognostic differences in the high‐ and low‐risk groups.

### 3.5 The nomogram of the combination of 9‐gene prognostic model and clinical factors

**FIGURE 7:**
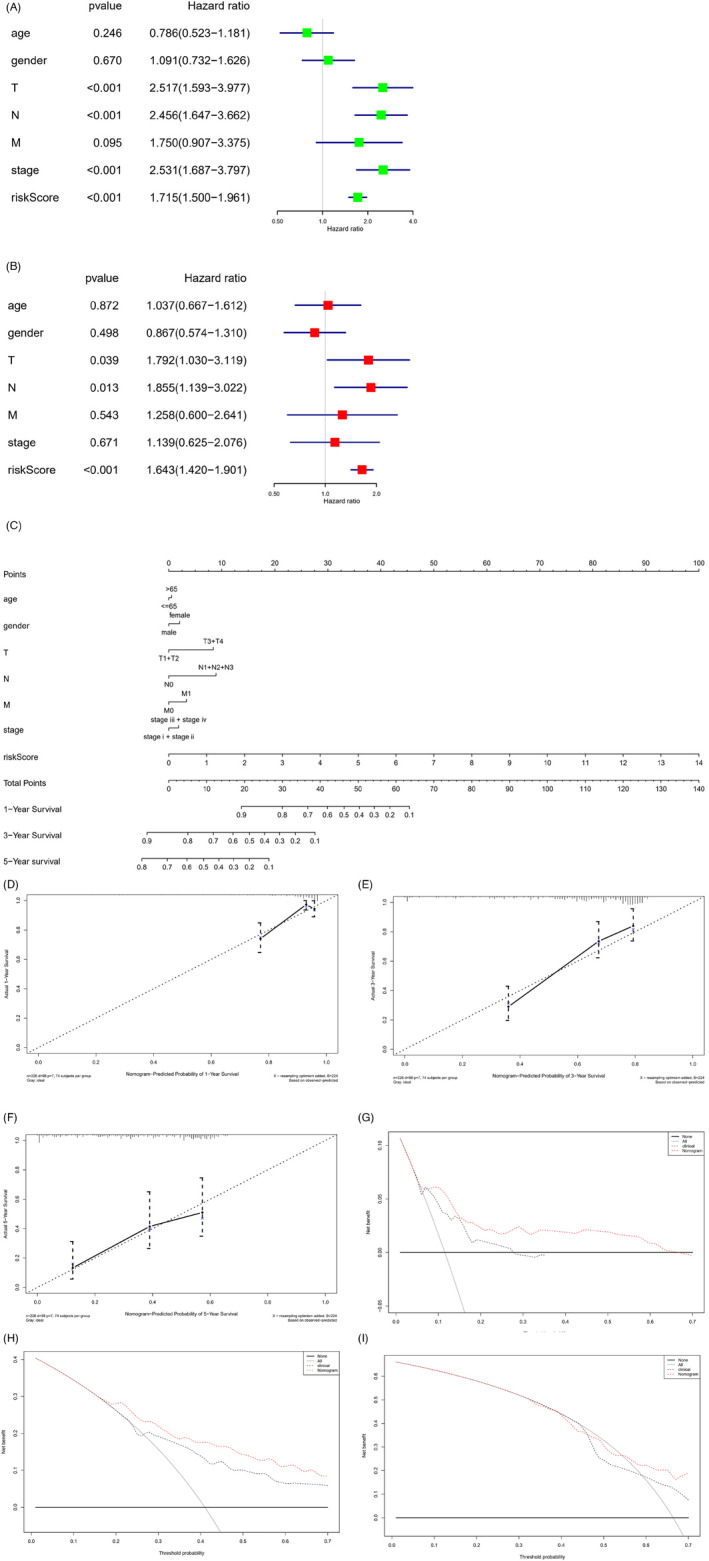
Construction of ITGB‐related nomogram and assessment of predictive ability. Forest map of (A) univariate Cox analysis and (B) multivariate Cox analysis on the risk score of 9‐feature gene model and clinical factors; (C) The constructed nomogram of risk score of 9‐feature gene and clinical factors; (D–F) Calibration curves of 1‐ (D), 3‐ (E), and 5‐year (F) survival of LUAD patients predicted by nomogram; (G–I) Decision curves of 1‐ (G), 3‐ (H), and 5‐year (I) survival of LUAD patients predicted by nomogram

Risk score of 9‐feature gene model could solely serve as a prognostic factor, and it could be combined with other clinical factors (age, sex, TNM stage, and tumor stage) to conduct univariate Cox analysis. The results demonstrated the notable significance of T, N stages, and risk score (*p *< 0.01), indicating that T, N stages, and risk score were closely correlated to LUAD prognosis (Figure 7A). Multivariate Cox analysis of these factors revealed that T, N stages, and risk score had evident significance (*p *< 0.05), while only risk score had extremely notable significance (*p *< 0.001) (Figure 7B). In conclusion, it was proved that the risk score obtained by the 9‐feature gene prognostic model could be utilized as a prognostic factor independent of traditional clinical features.

To better predict 1‐, 3‐, and 5‐year OS of LUAD patients, the risk score of 9‐feature gene prognostic model was combined with other clinical factors (age, sex, TNM stage, and tumor stage) to construct nomogram for predicting LUAD survival (Figure 7C). The calibration curve was applied to verify the consistency between the actual and predicted survival, reflecting the favorable fitting degree of the calibration curve (Figure 7D–F). The decision curve of the nomogram was then used to verify the predictive performance (Figure 7G–I). In summary, the constructed nomogram based on 9‐feature gene risk score, and clinical factors had a satisfactory predictive ability, which can provide help for clinicians to judge the prognosis of patients.

## 4 DISCUSSION

The development of sequencing technology accelerates the identification of more biomarkers and therapeutic targets, which has deepened the understanding of tumors. Nonetheless, it has been difficult to identify reliable biomarkers associated with LUAD treatment and prognosis. Several observations in recent years have demonstrated that the abnormal expression of integrin is closely associated with the progression of tumors.^18^, ^19^, ^20^ Therefore, LUAD was divided into subgroups based on the ITGB superfamily, and 9‐feature genes were screened from differentially expressed genes among different subgroups through the bioinformatics method, leading to the construction of an ITGB‐related prognostic model.

The feature genes used to establish the ITGB‐related prognostic model were FAM83A, AKAP12, PKP2, CYP17A1, GJB3, TMPRSS11F, KRT81, MARCH4 and STC1. FAM83A, AKAP12, PKP2, TMPRSS11F, KRT81, MARCH4, and STC1 were risk factors. FAM83A is located on chromosome 8q24 and is upregulated in LUAD, which is closely correlated to the poor prognosis of patients.^21^ A study uncovered that FAM83A can activate the expression of MAPK signaling pathway, thus enhancing the malignant progression of NSCLC.^22^ The high expression of FAM83A was revealed to induce a higher risk score in our study. AKAP12/Gravin is an A‐type kinase anchor protein. It was exhibited that AKAP12 is highly expressed in LUAD, promotes LUAD cell proliferation, migration, and invasion, and represses cell apoptosis.^23^ PKP2, a member of the p120ctn family of cell adhesion molecules, enhances cell proliferation, migration, and invasion by activating the EGFR signaling pathway in LUAD.^24^ PKP2 was elucidated to initially affect tumorigenesis, aggressiveness, malignant biological behavior, and immune infiltration of ovarian cancer.^25^ These results correspond to the risk ratio of the feature genes we analyzed, whereas the observation of TMPRSS11F and MARCH4 is less explored. KRT81, a hair keratin, has become a biomarker of breast cancer and was revealed to promote cancer cell migration and invasion.^26^ A study illustrated that STC1 is highly expressed in bladder cancer, enhances PD‐L1 expression, and increases the degree of T cell immune infiltration.^27^ The role of these feature genes in tumor progression has been elucidated, but their prognostic value has not been fully explored. The prognostic genes screened in this study are likely to be the biomarkers for LUAD treatment.

CYP17A1 and GJB3 are protective factors. CYP17A1 is a multifunctional hydroxylase of the cytochrome p450 family, which is expressed in the endoplasmic reticulum and adrenal cortex of testicular interstitial cells. It induces DNA demethylation to suppress cell proliferation, invasion, and metastasis of glioma.^28^ Meanwhile, bioinformatics analysis indicated that the low expression of CYP17A1 in the high‐risk group indirectly indicated the protective effect of CYP17A1. GJB3 has been less researched in previous studies. Collectively, the 9‐feature genes screened based on the ITGB superfamily in our study were not only related to LUAD prognosis but also used as a potential target for LUAD treatment.

The enrichment analyses of differentially expressed genes in high‐ and low‐risk groups indicated that these genes were primarily enriched in ECM interaction and cell cycle regulation. The adhesion between tumor cells and ECM is closely related to tumor invasion and metastasis. ECM is a dense network composed of structural proteins, adaptor proteins, proteoglycan, and enzymes, which exists in all tissue and mainly provide biochemical and structural support for tissue homeostasis. It provides support for cell adhesion and migration and regulates cell cycle progression.^29^, ^30^ ECM and its receptor integrin play a crucial role in tumor growth, invasion, metastasis, and drug resistance. As previously described, exosomes secreted by tumors express different integrin subtypes on their membranes, which are selectively absorbed by distant non‐tumor cells in a tissue‐specific manner, laying a preliminary foundation for tumor metastasis.^31^ Gan et al.^32^ uncovered that ECM 1 accelerates cell metastasis and glycolysis metabolism via inducing integrin β4/FAK/SOX2/HIF‐1α signaling pathway. Du et al.^33^ explored that integrin α3 activates c‐Src/extracellular signal in cervical cancer to modulate protein kinase/adherent plaque kinase signaling pathway and enhances tumor metastasis and angiogenesis, resulting in the unsatisfactory prognosis in patients with cervical cancer. Salemi et al.^34^ suggested that integrin α2β1 inhibits cell cycle to promote cell apoptosis and repress epithelial–mesenchymal transformation, so as to attenuate prostate cancer cell proliferation. In a word, the prognostic model constructed in this study was an ITGB‐related prognostic model, which reflected different prognostic results in two risk groups. The cause might be the modulation of integrin on ECM, cell–cell interaction, and intercellular signal transmission.

In conclusion, we utilized the NMF method to group LUAD samples according to the expression of ITGB superfamily genes, and subsequently constructed and evaluated the ITGB superfamily‐related prognostic risk assessment model based on the differentially expressed genes of ITGB‐related subgroups. Besides, the feature genes screened in this study might serve as the underlying targets for LUAD targeted therapy, which can provide references for LUAD prognosis determination. However, there are still some deficiencies in this study. This study is a pure retrospective study based on public datasets, while clinical samples should be contained to verify the predictive ability of the constructed model in the future.

## CONFLICT OF INTEREST

The authors declare that they have no potential conflicts of interest.

## AUTHOR CONTRIBUTIONS

YL contributed to the study design. GM conducted the literature search. LH, BW, ZP, and DS acquired the data. HY and CZ wrote the article. ZF performed data analysis. TZ drafted. GM and YL revised the article and gave the final approval of the version to be submitted. All the authors read and approved the final manuscript.

## PATIENT CONSENT STATEMENT

Not applicable and all authors consent to submit the article for publication.

## PERMISSION TO REPRODUCE MATERIAL FROM OTHER SOURCES

Not applicable.

## CLINICAL TRIAL REGISTRATION

Not applicable.

## Supporting information

Table S1

## Data Availability

The data used to support the findings of this study are included within the article. The data and materials in the current study are available from the corresponding author on reasonable request.
