# Supplementary material for: Construction of a prognostic risk assessment model for lung adenocarcinoma based on Integrin β family‐related genes
Source: J Clin Lab Anal. 2022 Apr 11;36(6):e24419. doi: 10.1002/jcla.24419 (PMC9169214; doi:10.1002/jcla.24419)
Supplement: Supplementary file 1 — Table S1 [file JCLA-36-e24419-s001.pdf]

gene	HR	z	pvalue
RHOV	1.205484	4.942623	7.71E-07
GGTLC1	0.899824	-4.92725	8.34E-07
PLCD3	1.34788	4.811335	1.50E-06
MYOZ1	0.822067	-4.72713	2.28E-06
PKP2	1.206925	4.684996	2.80E-06
DKK1	1.11774	4.618511	3.87E-06
NTSR1	1.110289	4.578397	4.69E-06
LYPD3	1.178344	4.55477	5.24E-06
FAM83A	1.20508	4.447914	8.67E-06
ABCC12	0.931977	-4.40046	1.08E-05
CREG2	1.154105	4.390937	1.13E-05
VAX1	1.065737	4.325541	1.52E-05
ABCC2	1.103584	4.090694	4.30E-05
KRT6C	1.064979	4.074084	4.62E-05
TNS4	1.127659	4.043955	5.26E-05
KRT6A	1.078511	3.980635	6.87E-05
KYNU	1.171153	3.979628	6.90E-05
CTSV	1.170745	3.961152	7.46E-05
FCER2	0.893195	-3.953	7.72E-05
MS4A1	0.887484	-3.91678	8.97E-05
PLA2G1B	0.914278	-3.88556	0.000102
FETUB	1.069751	3.851885	0.000117
KRT81	1.098834	3.836151	0.000125
IL1R2	1.171662	3.800241	0.000145
TMPRSS1[illegible]	1.065895	3.739056	0.000185
AKAP12	1.179634	3.736571	0.000187
MYBPHL	0.924786	-3.71272	0.000205
DNER	1.118566	3.654266	0.000258
KCNK17	0.866816	-3.65221	0.00026
GJB3	1.117914	3.642514	0.00027
CYP17A1	0.903895	-3.63284	0.00028
GFI1B	0.918293	-3.62996	0.000283
4-Mar	1.13977	3.611147	0.000305
AHNAK2	1.166732	3.580992	0.000342
TRIM7	1.168357	3.554636	0.000379
ACSM5	0.900155	-3.51789	0.000435
IGF2BP1	1.061563	3.507349	0.000453
GRAMD1[illegible]	1.164665	3.484385	0.000493
CD1C	0.876786	-3.45333	0.000554
SPOCK1	1.124376	3.453018	0.000554
SH2D5	1.112171	3.438359	0.000585
STC1	1.18004	3.436596	0.000589
SFTPC	0.947918	-3.40892	0.000652
HPGDS	0.861012	-3.39963	0.000675
SCGB3A1	0.931292	-3.39868	0.000677
CEACAM[illegible]	0.942073	-3.39607	0.000684
C1QL2	0.950055	-3.36721	0.000759
FCAMR	0.932598	-3.34097	0.000835
SPRR1B	1.048556	3.325931	0.000881
STAR	0.889991	-3.29716	0.000977
